# Investigating the borders of autism spectrum disorder: lessons from the former diagnosis of pervasive developmental disorder not otherwise specified

**DOI:** 10.3389/fpsyt.2023.1149580

**Published:** 2023-12-19

**Authors:** Romain Coutelle, Nathalie Coulon, Carmen M. Schröder, Olivier Putois

**Affiliations:** ^1^Hôpitaux Universitaires de Strasbourg, Department of Psychiatry, University of Strasbourg, Strasbourg, France; ^2^INSERM 1114, Strasbourg, France; ^3^TSA-SDI Expert Center and Psychosocial Rehabilitation Reference Center, Alpes Isère Hospital (Saint-Egrève Psychiatric Hospital), Grenoble, France; ^4^CNRS UPR 3212, Strasbourg, France; ^5^SuLiSoM UR 3071, Faculté de Psychologie, Université de Strasbourg, Strasbourg, France; ^6^Institut d’Immunologie et d’Hématologie, Institut Thématique Interdisciplinaire TRANSPLANTEX NG, Université de Strasbourg, Strasbourg, France

**Keywords:** autism spectrum disorder, pervasive developmental disorder, DSM-IV-TR, DSM-5, pathological demand avoidance, diversity, heterogeneity

## Abstract

**Introduction:**

Autism Spectrum Disorder (ASD) diagnosis is relatively consensual in typical forms. The margins of the spectrum and their degree of extension, however, are controversial. This has far-reaching implications, which extend beyond theoretical considerations: first, peripheral forms of autism are more prevalent than central forms; second, we do not know how relevant typical-targeted recommendations are for atypical forms. In DSM-IV-TR, these margins of autism were studied within the category of Pervasive Developmental Disorder – Not Otherwise Specified (PDD-NOS). In spite of its low reliability, this former diagnosis was of particular interest to shed light on the gray area of margins. The aim of this systematic is therefore to investigate the clinical characteristics of PDD-NOS in comparison with Autistic Disorder.

**Method:**

A stepwise systematic PRISMA literature review was conducted by searching PubMed and Web Of Science databases to select corresponding studies.

**Results:**

The systematic review included 81 studies comprising 6,644 children with PDD-NOS. Cross-sectional and longitudinal studies comparing PDD-NOS and AD showed that PDD-NOS corresponds to milder form of autism with less impact and less associated disorder, with the exception of schizophrenia and mood disorder.

**Discussion:**

Our review challenges initial views of PDD-NOS, and shows the clinical relevance of this diagnosis when dealing with the margins of autism, and the *de facto* diversity included in the spectrum. However, in view of the many limitations of PDD-NOS (low reliability, instability through time, low acceptability), we suggest taxonomic changes in DSM-5: we introduce a new category based on three main dimensions related to socialization impairment, emotional lability and psychotic symptoms.

**Conclusion:**

Our review argues for a distinction between AD and PDD-NOS on clinical characteristics and thus highlights the need to study the margins of autism. While the limitations of the PDD-NOS category made it irrelevant to investigate these margins from a research perspective, we believe that a multidimensional approach for mental health professionals taping socialization, emotion lability and psychotic symptoms would be interesting. Our review therefore encourage future studies to test relevant criteria for a new category and possibly identify developmental trajectories, specific interventions and treatments.

## Introduction

Since 2013 and the DSM-5, the diagnostic category of Autism Spectrum Disorder (ASD) has included 2 behavioral dimensions which represent the core defining features of ASD (1): (a) communication and social interaction deficits on the one hand, and (b) repetitive behaviors and restricted interests on the other hand (1). The term “ASD” suggests that the core features of the disorder can be measured dimensionally and, that they fall along a continuum of severity. No diagnostic subtypes are listed (1); instead, specifiers are provided to indicate associated dimensions, such as intellectual and/or language impairment, as well as the severity level of core ASD symptoms (2). Further, any known genetic or medical disorders are recorded and other co-occurring neurodevelopmental, mental, or behavioral disorders are indicated (2) to characterize subgroups.

In the DSM-5 (2), whatever the level of severity, even in the mildest forms, ASD symptoms are viewed as intrinsically autistic. However, to take one example, this conceptualization does not take into account the overlap between, on the one hand, some ASD symptoms, and on the other, Intellectual disability (3, 4), language impairment (5, 6), Attention Deficit/Hyperactivity Disorder (7, 8) or schizophrenia (9, 10). Moreover, mild ASD symptoms might be more akin to a natural variant of typical development than a formal disorder (11). Boundaries issues with typical development are also longitudinal, as has been shown in studies on “optimal outcome” or “Loss of Autism Diagnosis (LAD)” (12). These studies (13) focused on a group of individuals meeting criteria for ASD in childhood who no longer met them later in development. This definition entailed clear documentation of early ASD diagnosis, not meeting current diagnostic criteria (with ADOS scores similar to neurotypical peers with no history of ASD), and having overall cognitive, language, and social functioning standardized test scores within the average range (12). Confirmation that meeting criteria for ASD is not necessarily a lifelong state appears evident in the strikingly similar proportions (about 9%) of individuals experiencing LAD in prospective and retrospective studies (12). In line with the scope of our review, it is worth noticing that one study (14) showed that among ASD children included at age 2, those with PDD-NOS were significantly more likely than those with AD to move off the spectrum by age 4. ASD symptoms might be thus less specific than suspected in the DSM-5, especially in milder forms.

Envisioned in line with Kannner’s (15) first description and seminal work, autism is relatively consensual in its typical forms (full syndrome) (16). However the margins of the spectrum and their degree of extension are controversial (17) because of overlaps with other dimensions and blurred boundaries with typical development. This question is much more than a theoretical issue, since peripheral or “marginal” forms of autism might be more prevalent than central forms (see below). Furthermore, one could question the usefulness and relevance of recommendations made for typical forms when facing atypical ones. Past conceptualizations (18) contrasted the more prototypical form of autism, called “Autistic Disorder (AD),” with the margins of autism studied within the Pervasive Developmental Disorder - Not Otherwise Specified (PDD-NOS) category. Is therefore of special interest to explore these margins through the lens of this former diagnosis.

The PDD-NOS diagnosis category was first introduced in 1987 (19) to contrast with typical forms for autism labeled “Autistic Disorder” (AD). The DSM-III-R (19) states that *“this category should be used when there is a qualitative impairment in the development of reciprocal social interaction and of verbal and nonverbal communication skills, but the criteria are not met for autistic disorder, schizophrenia or schizotypal or schizoid personality disorder. Some people with this diagnosis will exhibit a markedly restricted repertoire of activities and interests, but others will not.”* In DSM-IV (20) and DSM-IV-TR (18), AD and PDD-NOS were included within the Pervasive Developmental Disorders (PDDs) that prelude ASD. PDDs also included Asperger Syndrome (AS), Childhood Disintegrative Disorder and Rett Syndrome (18, 20).

In DSM-IV-TR (18), the PDD-NOS category included atypical autism, i.e., presentations that did not meet the criteria for AD because of late age of onset, atypical symptomatology, or subthreshold symptomatology, or all of these. These recommendations echoed Towbin’s conceptualization (21) described in a reference textbook section published in 1997. He recommended the use of PDD-NOS in 4 indications: (a) as a temporary diagnosis when data are lacking or when the child is too young; (b) to designate a mild form of ASD; (c) in the case of late age of onset of autistic symptoms; (d) to depict a clinical picture with early symptom onset and impairment in social reciprocity (21). In terms of diagnostic use, the PDD-NOS, often envisioned as a diagnosis of subthreshold autism, seems to cover a wide range of clinical variability, sometimes referred to as heterogeneity; it would benefit from being studied in a more positive manner, by drawing on its clinical diversity (22).

Epidemiological studies show that at least half of PDD were PDD-NOS. In 2005, Fombonne (23) found a PDD-NOS prevalence of 37.1 per 10,000 for a PDD prevalence of 63 per 10,000 and an AD prevalence of 13 per 10,000. In 2010 in the United States, the results of the Centers for Disease Control and Prevention (CDC) showed a PDD prevalence of 14.6 per 1,000 in a population of 363,749 8-year old children (24). PDD-NOS were 46% of the PDD, AD 43% and AS 11%. Noticeably, between 2006 and 2010 the distribution of AD and PDD-NOS has not undergone substantial changes (24).

The diagnosis of PDD-NOS had a low inter-rater reliability. The inter-rater reliability was good to excellent (*k* from 0.95 to 0.67) for the distinction between PDD and non-PDD. However, the disentanglement of different subtypes of PDD was much less reliable (*k* from 0.18 to 0.65) (25–27). The DSM-IV-TR criteria were therefore more successful in differentiating PDD (25) from other psychiatric disorders than in distinguishing between the three main subtypes of PDD, namely AD, PDD-NOS and AS (28). In line with these results, a study (29) tried to assess the variation between behavioral phenotypes and clinical diagnoses of different autism spectrum disorders across 12 university-based sites. They found that clinical distinctions among categorical diagnostic subtypes of PDDs (AD, PDD-NOS and AS) were not reliable even across sites with well-documented fidelity, using standardized diagnostic instruments (29).

In spite of its low reliability, the former diagnosis of PDD-NOS is very interesting to study the margins of autism because of both its conceptualization at the end of the spectrum, and its high prevalence. The aim of our systematic review is thus to investigate the clinical characteristics of PDD-NOS in comparison with AD. The objectives were therefore to study comparatively (in both diagnoses) synchronic and diachronic characteristics such as autism symptoms, cognitive function, daily impact, associated mental health or physical health condition and developmental trajectory from infancy to adolescence. The comparison is intended to address the distinction between the typical form and the margins of autism as well as the relevance of the PDD-NOS/AD distinction. Our study therefore aims to determine whether this distinction helps mental health professionals to account for diversity within the spectrum, and to identify divergent trajectories, better than the current ASD category.

## Materials and methods

This study follows the Preferred Reporting Items for Systematic Reviews and Meta-analyses reporting guidelines (PRISMA) (30).

### Search strategy and selection criteria

A stepwise systematic literature review (30) was conducted by searching PubMed, MEDLINE, and Web Of Science databases for published peer-reviewed papers using the following keywords: “PDD-NOS” OR “Pervasive Developmental Disorder Not Otherwise Specified” from January first 1987 (the year of the DSM-III-R publication, introducing this diagnostic category for the first time) to June 16th 2022. The keywords were screened in the titles and abstracts.

### Eligibility criteria

#### Inclusion criteria

To be included in this review, the articles had to meet all following criteria: (a) include children and/or adolescents (31), (b) compare toddlers, children or adolescents with PDD-NOS with children with AD (c) be published in English, (d) use a quantitative design. RC and OP applied the eligibility criteria and screened the records to select included studies.

#### Exclusion criteria

The following exclusion criteria were used: (a) no reviews, comments or clinical cases, (b) methodological features: number of participants included in the AD or the PDD-NOS groups ≤10, adults included only, lack of comparison group, comparison with a group different from AD (e.g., AS), participants whose diagnosis has been confirmed by a classification other than the DSM-III or the DSM-IV, test validation studies, (c) epidemiological data in order to focus on mostly clinical well-characterized populations, (d) therapeutic trials, (e) anatomical, biological or fMRI studies (isolated, contradictory and/or non-replicated results) are (difficult to interpret), (f) language other than English, and (g) qualitative studies.

### Outcome measures and data extraction

The following variables were extracted: First author (second, third author and journal if needed to distinguish from other studies); Year of publication; Sample Size; Age (mean ± SD); Sex (% Males); Functioning level when available and when age at inclusion (at baseline in longitudinal studies) > 6 y.o. (to assess comorbid ID); Objective; Design of the Study; Methods (only with longitudinal studies); Main assessment tools; Results.

### Quality assessment

All included studies were observational, with a similar comparative framework. Quality assessments in such observational studies is controversial, with no clear agreement on rating methods (32). We therefore did not assess quality.

## Results

### Database

Our search from 1987 to 2022, undertaken on June 16th 2022, found 510 articles on PubMed and 431 on Web of Science. After manually removing all duplicates, 681 references remained. Based on their titles and abstracts, 256 papers were excluded for lack of relevance as they displayed no separate groups for AD and PDD-NOS. Most of these articles focused on PDDs (not PDD-NOS), ASD or grouped PDD-NOS, AD and/or AS into common groups. Other articles studied PDD-NOS in specific groups such as X-Fra, cerebral palsy, Down Syndrome... Our search strategy yielded 425 full-text articles assessed for eligibility. After conducting a full-text analysis of all these papers and excluding those not meeting our inclusion criteria, we ended up with 81 relevant studies, 67 of which were cross-sectional and 14 longitudinal.

### Studies

A total of 6,644 children with PDD-NOS and 11,156 children with AD were included. 47 studies were from the United States (58%), 12 from Netherlands (15%), 5 from Turkey (6%), 3 from Italy (4%), and 3 from Sweden (4%) The mean age in each group (when available) was 5.85 and 5.84 years, respectively. The mean age was low because many studies included toddlers or/and pre-schoolers with PDD-NOS (52%). Sex was not systematically specified in each group. Based on available data, PDD-NOS groups comprised 75.1% of males. IQ was not always specified, in particular in studies in toddlers or preschoolers. Based on available IQ measures, average IQ was 81.93.

**Figure 1:**
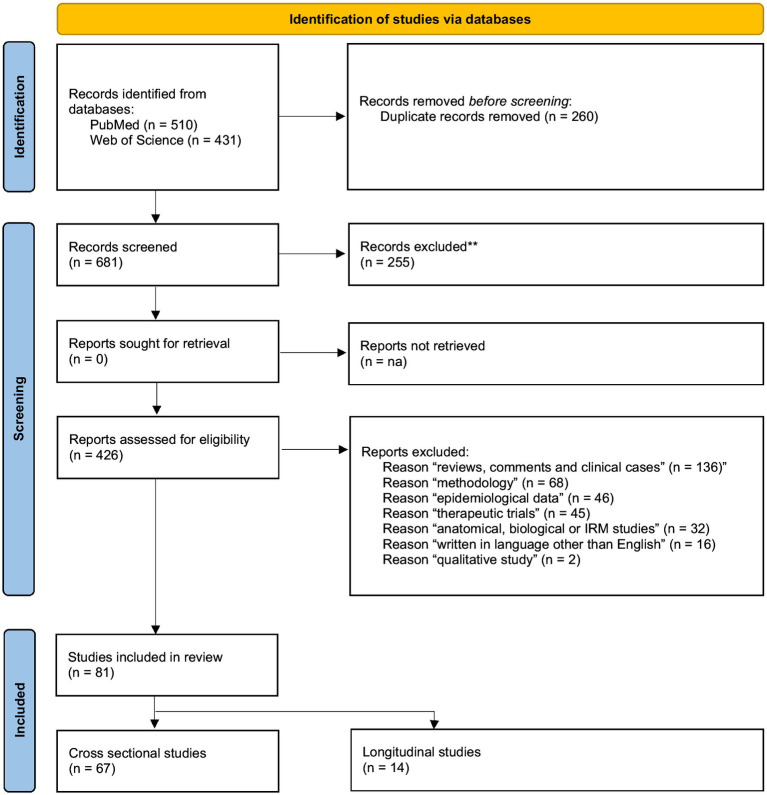
PRISMA flowchart.

Within the 67 selected cross-sectional studies, we distinguished those relating to (a) autism symptoms (22 studies), (b) perinatal, developmental and functional aspects (31 studies), (c) associated mental health conditions (14 studies) and (d) associated physical health conditions (6 studies). We chose these categories in part because they best served our goal, of investigating the clinical characteristics (autism symptoms, associated conditions/dimensions, developmental trajectories and adaptive behavior) of PDD-NOS in comparison with AD, and in part because they were those that best matched selected studies. All cross-sectional studies could be included in these three categories. As explained above, 14 longitudinal studies were also selected (Figure 1).

The total number of the studies presented in the corresponding tables (Tables 1–5) is 87 and not 81 because Carigi et al.’s study informed the 4 domains, and was thus selected in the corresponding categories (33). Walker et al. study informed 2 domains (34). Finally, Mandy et al.’s study informed 3 domains (35).

**Table 1:** Autism symptoms in PDD-NOS in comparison of AD.

Authors	Year	Population		Age	Males in PDD-NOS group		Country	Functioning level	Objective	Study design	Main assessment tools	Results
		AD	PDD-NOS		*N*	%						
Carigi et al.	2014	46	71	AD: 67.78 months (39.59)^1^ PDD-NOS: 45.20 months (17.28)	56	79	Italy	DQ^2^: 59.86 (15.42)	To identify clinical features that might prove able to differentiate AD from PDD-NOS	Comparative study	The ADI-R^3^ The ADOS^4^ The CARS^5^ The Griffith’s Mental Development Scale	Higher scores were rated on the ADI-R (with the exception of restrictive and repetitive behavior), on the ADOS and on the CARS in AD in comparison with PDD-NOS.
Mahoney et al.	2014	72	105	AD: 53.12 months (18.31) PDD-NOS: 52.86 months (18.47)	86	82	USA		To characterize and compare social interaction profiles of young children with AD and PDD-NOS	Comparative study	The Screen for Social Interaction	Children with AD and those with PDD-NOS had similar social interaction profiles
Joseph et al.	2013	128	46	AD: 4.14 years (1.57) PDD-NOS: = 4.14 (1.57)	41	89	USA	Non-Verbal Developmental Quotient: 80.37 (17.60)	To investigate restricted, repetitive and stereotyped patterns of behavior, interests and activities in children with AD and those with PDD-NOS	Comparative study	The Repetitive Behavior Scale-Revised	The AD group did not differ from PDD-NOS on any subscale (Self-Injurious Behavior, Stereotyped Behavior, Compulsive Behavior, Ritualistic/Sameness Behavior and Restricted Behavior).
Matson et al.	2013	115	113	26.63 months (5.22)			USA		To compare ASD symptoms in children with AD and those with PDD-NOS	Comparative study	the BISCUIT^6^-Part 1	The AD group had higher rates of ASD symptoms than the PDD-NOS group in all three domains (repetitive behaviors, social interaction and communication)
Kozlowski et al.	2012	20	20	8.37 years (3.22)	13	65	USA	No ID	To determine differences between AD and PDD-NOS on appropriate and inappropriate social skills	Comparative study	The Matson Evaluation of Social Skills in Youngsters-II	Social skills did not differ between children with AD and PDD-NOS
Falck-Ytter et al.	2012	40	25	AD: 2107 days (339) PDD-NOS: 2224 days (252)			Sweden	Total IQ: 84.15 (10.53)	To compare gaze performance in children with AD and those with PDD-NOS	Comparative study	Eye tracking The Wechsler Preschool and Primary Scale of Intelligence – Third Edition.	Gaze performance did not significantly differ between the AD and the PDD-NOS group
Hattier et al.	2011	197	197	AD: 26.59 months (4.75) PDD-NOS: 25.54 (4.48)	143	73	USA		To examine whether the AD group would have significantly greater levels of impairment in communication and socialization than the PDD-NOS	Comparative study	The BISCUIT-Part1 The Battelle Developmental Inventory, 2nd Edition	In regards to communication, no significant differences were found between children with AD and PDD-NOS. For socialization, the comparison AD vs. PDD-NOS were found to differ significantly
Karabekiroglu	2012	47	94	AD: 4.1 (2.0) PDD-NOS: 5.4 (2.4)	71	76	Turkey		To investigate differential features of PDD-NOS such as presenting symptoms, developmental history, and comorbidity with respect to autism	Comparative study	The K-SADS^7^ The First Clinical Admission Questionnaire and Clinical Assessment Form Preliminary PDD-NOS symptom screening scale	The most common presenting symptoms in the PDD-NOS and AD groups are reported significantly less in prevalence in the PDD-NOS group
Mandy et al.	2011	97	66	AD: 9.43 years (3.50)^2^ PDD-NOS: 9.05 years (3.51)	52	79	UK	Verbal IQ: 87.60 (15.98)	To characterize a group of individuals diagnosed according to a clearly operationalized DSM-IV-TR definition of PDD-NOS	Comparative study	The Developmental, Dimensional and Diagnostic Interview The ADOS	The AD and PDD-NOS groups did not differ significantly according to reciprocal social interaction or social communication measures. The PDD-NOS group had less repetitive, stereotype behaviors than the AD group.
Sipes et al.	2011	270	270	17 to 34 months			USA		To examine the effects of a diagnosis of ASD (AD or PDD-NOS) and motor skills impairments and their interaction on social skills impairments	Comparative study	The Biscuit-part 1 The Battelle Developmental Inventory–Second Edition	Those with AD had significantly higher (i.e., more impaired) social skill impairments than those with PDD-NOS. Level of fine motor skills affected social skills more so in those with AD than in those with PDD-NOS
Fein et al.	2010	15	13	10.42 years (2.2)			USA		To compare the rates of contagious yawning in a group of children with AD and PDD-NOS	Comparative study	A contagious yawning experiment	Children diagnosed with AD were less likely to exhibit contagious yawning than children diagnosed with PDD-NOS
Horovitz et al.	2010	220	220	AD: 27.22 months (4.74) PDD-NOS: 26.44 (4.77)	156	71	USA		To compare communication impairments of toddlers diagnosed with AD and PDD-NOS	Comparative study	The BISCUIT-Part 1	Toddlers with AD had more total communication impairments than did toddlers with PDD-NOS
Fodstad et al.	2009	161	140	26.53 months (5.02)			USA		To determine the nature and rate of verbal and non-verbal communication as well as social skills	Comparative study	The BISCUIT part 1	Deficits in communication and social skills were more obvious and pronounced in those with AD, then PDD-NOS
Matson, Dempsey & Fodstad (*Developmental Neurorehabilitation*)	2009	140	121	26.63 months (5.12)			USA		To determine the nature and rate of stereotypies and ritualistic behaviors in a population of infants with AD or PDD-NOS	Comparative study	The BISCUIT	Stereotypies and repetitive/ritualistic behaviors were most common in AD, then PDD-NOS
Matson, Dempsey & Fodstad (*Journal of Developmental and Physical Disabilities*)	2009	69	34	7.99 years (3.25)			USA		To distinguish between AD and PDD-NOS To evaluate specific diagnostic profiles within and across ASD is also critical	Comparative study	The Autism Spectrum Disorders-Diagnostic for Children	Severity of impairment was greatest for AD, followed by PDD-NOS. AD and PDD-NOS differed on Social Relationships and Verbal Communication but not on Insistence on Nonverbal Comm./Socialization, Sameness/Restricted Interests
Matson, Fodstad & Dempsey	2009	171	144	26.50 (4.97)			USA		To distinguish between a diagnosis of AD from PDD-NOS in a sample of infants and toddlers	Comparative study	The BISCUIT-Part 1	The study identified 11 items that distinguished young children with autism from those with PDDNOS. This items were engages in repetitive motor movements for no reason, use of language in conversations, shares enjoyment with others, interest in participating in social activities, restricted interest and activities, sticking to purposeless odd routines or rituals, abnormal preoccupation with object parts, reads non-verbal cues of others, use of non-verbal communication, abnormal of repetitive hand or arm movements and development of social relationships
Chawarska et al.	2007	51	24	AD: 28.5 months (4.7) PDD-NOS: 27.8 months (5.7)			The ADI-R	Mullen visual reception DQ: 84 (16)	To examine factors that influence the timing of parental recognition of developmental problems including severity of autistic symptoms	Comparative Study	The ADI-R The Mullen Scales of Early Learning	The mean age of onset of parental concerns in the group with AD was comparable to that reported in the PDD-NOS group. Parents of children with AD were significantly more likely to report their children as having medical problems and motor delays, as well as unusual autistic-like stereotyped behaviors than parents of children with PDD-NOS. Children with PDD-NOS had more non-specific problems related to sleeping, eating, and activity level
Verté et al.	2006	57	31	AD (without ID): 8.8 years (1.8) PDD-NOS: 8.8 years (1.6)	26	84	The Netherlands	Full Sclale IQ: 98.0 (14.7)	To explore whether children with AD (without ID) and PDD-NOS can be differentiated in terms of their Children’s Communication Checklist profile	Comparative Study	The Children’s Communication Checklist	The AD (without ID) group had more problems of coherence than the PDD-NOS groups. The AD (without ID) group had lower conversational rapport scores than the PDD-NOS Group. The PDD-NOS group had fewer restricted interests than the HFA and AS groups. The HFA group had a lower average pragmatic composite score than the PDD-NOS group
Walker et al.	2004	216	21	AD: 86.3 months (38.0) PDD-NOS: 99.0 months (71.8)	18	86	USA	Leiter IQ: 82.38 (33.40)	To describe the characteristics of children with PDD-NOS, diagnosed using a consensus best estimate	Comparative study	The ADI-R The ADOS The ABC^8^ The Leiter International Performance Scale	Children with PDD-NOS have fewer autism symptoms than children with AD.
Buitelaar et al.	1999	205	80	Pre-school children to young adults	69	86	The Netherlands	ID in 25% of cases	To explore the boundaries of PDD-NOS vis à vis AD	Comparative study	ICD 10/DSMIV criteria of AD and PDD-NOS	The three items with the strongest differentiating power were (a) lack of varied spontaneous make-believe play, (b) preoccupation with restricted patterns of interest, and (c) impairment of play before age 3 years
Klin et al.	1999	34	34	AD: 7.37 years (2.93) PDD-NOS: 6.59 years (2.01)			USA	Non-Verbal Mental Age: 5.19 years (1.00)	To explore whether or not any deficits found in face recognition applied to AD and PDD-NOS	Comparative study	The K-ABC	Children with AD performed significantly worse on face recognition than the PDD-NOS group
Mayes et al.	1993	40	40	AD: 6.9 years (5.4) PDD-NOS: 6.6 years (4.8)	33	75	USA	Mental age: 3.74 (2.60)	The delineation, of specific clinical features that differentiate PDD-NOS from AD	Comparative study	24 items selected from the DSM III-R criteria for AD, the ICD-10 Draft Research Criteria for childhood autism, the ABC and the VABS	Items that distinguished AD with PDDNOS from those with autism related to the degree of socialization and relatedness with children with PDD-NOS showing less severe disturbances in relatedness

^1^(SD). ^2^Developmental Quotient. ^3^Autism Diagnostic Interview -Revised. ^4^Autism Diagnostic Observation Schedule. ^5^Child Autism Rating Scale. ^6^Baby and Infant Screen for Children with aUtIsm Traits. ^7^Schedule for Affective Disorders and Schizophrenia for School-Age Children. ^8^Vineland Adaptive Behavior Scale. ^9^Autistic Behavior Checklist.

**Table 2:** Perinatal, developmental and functional aspects in PDD-NOS in comparison of AD.

Authors	Year	Population		Age	Males in PDD-NOS group		Country	Functioning level	Objective	Study design	Main assessment tools	Results
		AD	PDD-NOS		*N*	%						
Carigi et al.	2014	46	71	AD: 67.78 months (39.59)^1^ PDD-NOS: 45.20 months (17.28)	56	79	Italy	DQ^1^: 59.86 (15.42)	To identify clinical features that might prove able to differentiate AD from PDD-NOS	Comparative study	The CGI^2^ The Griffith’s Mental Development Scale The Health Related Quality of Life Questionnaire	Absence of pointing, were more frequent in the AD group. The impact on patients and families were also greater in the AD group.
Hill et al.	2014	40	44	6 to 13 years			USA	Verbal Comprehension/Verbal IQ and Processing Speed composite scores: 158.18 (22.408)	To examine the potential moderating effects of anxiety and cognitive functioning on the relation between local processing and social skills in children with ASD	Comparative study	The Wechsler Preschool and Primary Scale of Intelligence – Third Edition. The Wechsler Intelligence Scale for Children – Fourth Edition The Anxiety and Social Skills subscale scores from the Parent Report Form of the Behavioral Assessment System for Children – Second Edition	The Block Design subtest scores did not differ between children with AD and those with PDD-NOS. Anxiety and cognitive functioning moderated the association between local processing and social skills whatever the diagnosis considered
Thurm et al.	2014	125	42	AD: 48.6 months (16.9) PDD-NOS: 48 months (15.7)	35	83	USA	DQ: 72.5 (19.9)	To provide novel data on timing information about both attainment and any loss of skills to capture onset patterns in young children	Comparative study	A detailed caregiver interview	In children with AD, pointing to express interest and show object were lesser frequently attained skills than in those with PDD-NOS. The percentage of children reported to have lost at least one skill revealed that the AD group had the largest percentage of children with caregiver report of any loss followed by PDD-NOS
Kose et a.	2013	46	38	AD: 8.6 years (3.8) PDD-NOS: 6.3 years (2.5)	32	84	Turkey		To investigate the Health Related Quality of Life of children within ASD groups.	Comparative study	The Pediatric Quality of Life Inventory 4.0 scored by mothers	Psychosocial, social, school functioning and total summary score of the AD group were lower than PDD-NOS
Peters-Scheffer et al.	2013	87	24	AD: 5.58 years (17.19) PDD-NOS: 5,92 years (19.88)			The Netherlands	IQ: 32.85 (14.42)	To compare behavioral flexibility in those with AD and those with PDD-NOS	Comparative study	The Behavior Flexibility Rating Scale – Revised The Mullen Scales of Early Learning	No differences in behavioral flexibility toward persons were found between children with PDD-NOS plus ID and children with AD plus ID
Schlooz et al.	2013	15	21	AD: 131.27 months (13.59) PDD-NOS: 130.29 (16.73)	21	100	The Netherlands	Full Scale IQ: 109.43 (12.16)	To test whether the AD group reach higher scores on the Embedded Figures Test than the PDD group	Comparative study	The Children’s Embedded Figures Test The Adult Embedded Figures Test	Children and adolescents with AD and PDD-NOS did not differ on visual-perceptual tests
Visser et al.	2013	121	75	AD: 32.6 months (6.6) PDD-NOS: 34.6 months (5.8)			The Netherlands		This study compared the occurrence of pre- and perinatal risk factors between the narrowly (AD) versus broadly defined autistic phenotypes (PDD-NOS)	Comparative study	A parental questionnaires derived from the Prechtl optimality scales with addition of relevant items like paternal age, intoxications and maternal stress	Cases with PDD-NOS differed from those with AD by higher exposure to smoking during pregnancy and by a negative association of smoking with IQ. SDP appears to contribute more to broadly defined than to narrowly defined autistic phenotypes
Blijd-Hoogewys et al.	2012	35	65	11.23 years (3.35)			The Netherlands	Performance IQ: 91.98 (19.26)	To examine whether different ASD subtypes (Autistic disorder and PDDNOS) show different profiles	Comparative study	The Behavior Rating Inventory of Executive Functions	The BRIEF scale scores for AD and PDDNOS do not differ significantly on executive funtions
de Bildt et al.	2012	60	95	11.90 years (3.92)			The Netherlands	ID	To examine the rates of Visual Rooting Reflex in the AD and PDD-NOS subgroups	Comparative study	The ADOS^3^ The VABS^4^	Children with AD + ID displayed a Visual Rooting Reflex more often than children with PDD-NOS + ID
Kjellmer et al.	2012	22	44	69 months (8.5)			Sweden	Full Scale IQ: 86.3 (11.3)	To compare language comprehension between children with AD and those with PDD-NOS	Comparative study	The Comprehension Scale of the Reynell Developmental Language Scales III	Children with AD and PDD-NOS did not differ in language comprehension
Mandy et al.	2011	97	66	AD: 9.43 years (3.50)^2^ PDD-NOS: 9.05 years (3.51)	52	79	UK	Verbal IQ: 87.60 (15.98)	To characterize a group of individuals diagnosed according to a clearly operationalized DSM-IV-TR definition of PDD-NOS	Comparative study	The Developmental, Dimensional and Diagnostic Interview The ADOS	For gross motor and fine motor impairment there were no group differences. By contrast, on measures of visuo-spatial impairment and auditory sensitivity the PDD-NOS group were less impaired that the AD group
O’Donnell et al.	2012	28	14	36 to 59 months			USA		To explore the sensory processing differences between two subgroups of these children (AD and PDD-NOS)	Comparative study	The Short Sensory Profile	Short Sensory Profile scores between the two subgroups of children (AD and PDD–NOS) did not significantly differed
Beeger et al.	2010	11	20	AD: 9,33 years (1.67) PDD-NOS: years 9,17 (1.75)			The Netherlands	Full Scale IQ: 96.1 (11.1)	To test whether children with AD and PDD-NOS differ in their tendency to account for the impact of an emotionally charged initial situation in their emotional reaction to a successive situation (emotional transfer).	Comparative study	Two parallel sets of four stories	Children with AD reported equal effects of preceding positive and negative emotions, and children with PDD-NOS were relatively unaffected by the preceding emotions
Huemer et al.	2010	171	119	AD: 10.41 years PDD-NOS: 10.08 years	91	76	USA		To compare decoding and comprehension in children with AD and those with PDD-NOS	Comparative study	The Woodcock reading mastery test—revised Slosson oral reading test-revised Gray oral reading test-revised, 4th edition Lindamood auditory conceptualization test The Peabody picture vocabulary test third edition Detroit tests of learning aptitude-4th edition, word opposites Detroit tests of learning aptitude-2nd edition, Oral directions	Group differences on decoding and comprehension between the AD and the PDD-NOS group were not significant
Matson, Mahan, Fodstad et al.	2010	117	112	AD: 26.83 months (4.78) PDD-NOS 25.76 months (4.48)	80	71	USA		To analyze the differences between motor skills in infants and toddlers with various AD and PDD-NOS	Comparative study	The Battelle Developmental Inventory, 2nd Edition	Toddlers with PDDNOS did not have significantly different fine or gross motor skills than toddlers with AD
Matson, Mahan, Kozlowski et al.	2010	165	166	AD: 27.52 months (4.61) PDD-NOS: 26.64 months (4.69)	123	74	USA		To examine whether there are differences in age of onset for developmental milestones, such as saying one’s first word, first phrase, onset of crawling and onset of walking, between the AD and the PDD-NOS group	Comparative study	The BISCUIT^5^ demographic form	The children with AD began crawling significantly later than those with PDD-NOS. No other comparison was significant
Zander et al.	2010	85	94	AD: 10.22 years (2.16), PDD-NOS: 10.73 years (2.15)	51	54%	Sweden		To examine index score profiles and their characteristics in a large Swedish sample consisting of children with AD and PDD-NOS	Comparative study	The Swedish version of the Weschler Intelligence Scale for Children – third edition	There were no significant differences between children with AD and those with PDD-NOS on any index score of the Weschler Intelligence Scale for Children
Dawson et al.	2009	314	84	4 to 19 years			Australia		To investigate the association between birth defects and ASD for AD and PDD-NOS	Comparative study		Odds ratios for birth defects were similar for AD and PDD-NOS
Meilleur et al.	2009	80	44	6.3 years (4.1)			Canada		To compare regressive and non-regressive ASD children for their profile of diagnosis	Comparative study	The ADI-R	Children with AD were significantly more likely to regress in skills other than language when compared with children with PDD-NOS
Perry et al.	2009	192	66	51.70 months (12.51)			Canada		To examine whether the adaptive behavior of children with AD differ from individually matched children with PDD-NOS	Comparative study	The VABS	No differences were found between matched pairs of children with AD and PDD-NOS on adaptive behavior
Portoghese et al.	2009	21	25	AD:3.2 years (1.04) PDD-NOS: 3.3 years (0.92)	25	92	Italy		To discern differences between preschool children with AD and children with PDD-NOS as regards their developmental and behavioral levels	Comparative study	The Revised Psychoeducational Profile	All behavioral areas and almost all developmental domains (with the exception of the cognitive verbal) assessed were more severely impaired in the AD group.
de Bruin et al.	2006	13	76	AD: 8.62 years (1.81) PDD-NOS: 8.39 years (1.86)	65	86	The Netherlands	Full scale IQ: 89.58 (19.38)	To compare VIQ–PIQ and subtest patterns in children with AD from children with PDD-NOS	Comparative study	The WICS-R	No overall differences between VIQ and PIQ were found in PDD-NOS and autism.
Verté et al.	2006	50	25	AD: 8.7 years (1.9) PDD-NOS: 8.5 years (1.4)	20	80	The Netherlands	Full scale IQ: 98.3 (14.4)	To investigate whether children with AD (without) and PDDNOS can be differentiated from each other on their neurocognitive executive functioning (EF) profile	Comparative study	The change task The circle drawing task Test of Everyday Attention for Children, Subtest Opposite Worlds The Self-Ordered Pointing Task, Abstract Designs The tower of London The Wisconsin Card Sorting Test An adaptation of the Controlled Word Association Task	The executive function profile of the PDDNOS group was less disturbed than the profile of the AD group (higher Visual Working Memory and Planning scores).
Glason et al.	2004	314	84	4 to 19 years			Australia		To examine the association of obstetric factors with autism spectrum disorders for a cohort of children, using obstetric data contained in a statutory database collected at the time of birth	Comparative study		The PDD-NOS group had similar types of complications to the autism group, but some variables reached statistical significance. These variables were greater frequencies of threatened abortion, a labor duration of less than 1 h. Cases were more likely to have experienced cephalopelvic disproportion, been delivered by an elective or emergency cesarean section.
Rhea et al.	2004	20	20	AD: 6.5 years (1.8) PDD-NOS 6.2 years (1.6)	18	90	USA	IQ: 76.3 (16)	To provide a microanalysis of differences in adaptive functioning seen between well-matched groups of school-aged children with autism and those diagnosed as having PDD-NOS	Comparative study	The VABS	Findings indicate that children with PDD-NOS scored higher in expressive communication; specifically, the use of elaborations in syntax and morphology and in pragmatic use of language to convey and to seek information in discourse
Walker et al.	2004	216	21	AD: 86.3 months (38.0) PDD-NOS: 99.0 months (71.8)	18	86	USA	Leiter IQ: 82.38 (33.40)	To describe the characteristics of children with PDD-NOS, diagnosed using a consensus best estimate	Comparative study	The VABS The Leiter International Performance Scale	Children with PDD-NOS have higher functioning levels than children with AD.
Allen et al.	2001	176	18	AD without ID: 57.8 months (15.2) AD with ID: 59.6 months (16.4) PDD-NOS without ID: 56.0 months (13.3) PDD-NOS with ID: 66.0 months (15.15)	14	78	USA	ID in 90% of cases	To determine whether preschool children having AD differed in kind from those of with PDD-NOS	Comparative study	The VABS The Wing Autistic Disorder Interview Checklist The Stanford-Binet 4^th^ edition	PDD-NOS children did not differ significantly from the AD children in verbal and adaptive skills. The PDD-NOS did also not differ from the AD children in maladaptive behaviors.
Juul-Dam et al.	2001	61	16	5 years			USA		To examine various pre-, peri-, and neonatal factors	Comparative study	Risk factors assessment	The PDD-NOS group showed results similar to those of the AD group, with significantly lower increased incidence of hyperbilirubinemia
Buitelaar et al.	1999	20	20	AD: 12.5 years (3.2) PDD-NOS: 12.4 years (3.1)	17	85	The Netherlands	Full Scale IQ: 98.7 (18.3)	To explore wether autistic children without ID and autistic-like (PDD-NOS) children could be distinguished in social cognitive performance	Comparative study	First order ToM tasks Second order ToM tasks Emotion recognition	AD and PDD-NOS children could not be significantly differentiated from each other on social cognitive performance
Ghaziuddin et al.	1998	12	12	AD: 10.3 years (2.9) PDD-NOS: 10.1 years (2.7)	10	83	USA	Full IQ: 78.2 (14.5)	To assess the presence of clumsiness in patients with AD or PDDNOS	Comparative study	The Bruininks Oseretsky test	Patients with PDD-NOS were found to be less impaired on battery test scores than those with AD
Volkmar et al.	1993	199	74	AD: 13.29 years (7.64) PDD-NOS: 7.47 (6.05)	58	78	USA	IQ:70.23 (22.46)	To explore the nature of sex differences in AD and PDD-NOS	Comparative study	The ABC The VAB	The AD males were 8.8 times more likely than females to have full-scale IQ scores >70; whereas in the PDD-NOS group males were only 1.5 times more likely to have full-scale IQs > 70

^1^(SD). ^2^Clinical Global Impression Severity Scale. ^3^Autism Diagnostic Observation Schedule. ^4^Vineland Adaptive Behavior Scale. ^5^Baby and Infant Screen for Children with aUtIsm Traits.

**Table 3:** Associated mental health conditions in PDD-NOS in comparison with AD.

Authors	Year	Population		Age	Males in PDD-NOS group		Country	PDD-NOS Functioning level	Objective	Study Design	Methods and main assessment tools	Results
		AD	PDD-NOS		*N*	%						
Chien et al.	2020	2,929	461	AD: 5.6 years (10.6)^1^ PDD-NOS: 12.7 years (16.5)	311	68	Taiwan	Absence of ID	To investigate the incidence of comorbid Schizoprenia Spectrum Disorder, Bipolar Disorder, Major Depressive Disorder and ASD associated neurodevelopmental conditions in AD and PDD-NOS using a national insurance database	Comparative study	Claim records completed by physicians	Patients with PDD-NOS had a higher risk for developing Schizoprenia Spectrum Disorder, Bipolar Disorder, Major Depressive Disorder than AD.
Carigi et al.	2014	46	71	AD: 67.78 months (39.59) PDD-NOS: 45.20 months (17.28)	56	79	Italy	DQ^2^: 59.86 (15.42)	To identify clinical features that might prove able to differentiate AD from PDD-NOS	Comparative study	Semi-structured interview to collect information about the developmental history	Pharmacotherapy were more frequent in the AD group.
Joshi et al.	2014	ASD clinic: 89 Psychiatry clinic: 25	ASD clinic: 30 Psychiatry clinic: 192	3 to 17 years			USA		To investigate whether phenotypes of ASD and associated psychopathology and dysfunction varied by referral source. To this end, youth with ASD attending a specialty clinic for ASD were compared to those attending a general psychiatry clinic	Comparative study	The K-SASD^3^ The DSM Global Assessment of Functioning Scale	More ASD clinic youth met criteria for AD; more youth referred to the psychiatry clinic met criteria for PDD-NOS. General psychiatry clinic youth with ASD suffered from a greater burden of psychopathologies (Oppositional defiant disorder, Major Depressive Disorder and psychosis) higher levels of dysfunction and more pharmacotherapy.
Kozlowski et al.	2012	92	114	AD: 26.57 months (4.84) PDD-NOS: 26.14 months (5.036)	57	50%	USA		To identify differences in challenging behavior endorsement rates between AD and PDD-NOS groups	Comparative study	The BISCUIT^4^-Part 3	Individuals with AD endorse significantly greater amounts of challenging behaviors (aggressive/disruptive behaviors, stereotypic behaviors, and self-injurious behaviors) than individuals with PDD-NOS
Gjevik et al.	2011	47	12	11.8 years (3.3)			Norway		To assess the prevalence of current comorbid DSM-IV disorders in a special school population of children and adolescents with AD and PDD-NOS	Comparative study	The K-SADS	Obsessive-Compulsive Disorder and Conduct Disorder was more prevalent in children with PDD NOS than in those with AD.
Horovitz et al.	2011	291	241	26.33 months (4.76)	168	70	USA		to examine the differential endorsement of challenging behaviors with respect to diagnosis subtype (AD vs. PDD-NOS)	Comparative study	The BISCUIT part 3	Young children with AD are endorsed for engaging in significantly more challenging behaviors than those with PD-NOS (Destructive/aggressive behavior, stereotypic behaviors but not self-injurious behavior)
Sipes et al.	2011	247	211	AD: 27.21 months (4.84) PDD-NOS; 26.47 months (4.84)	152	72	USA		To determine if tantrum/conduct problems were rated more frequently in toddlers with AD than in those with PDD-NOS	Comparative study	The BISCUIT-Part 2	Children with AD exhibited greater symptoms of tantrum/conduct problems
Snow et al.	2011	54	54	AD: 65.6 months (28.7) PDD-NOS: 65.4 months (29.7)			USA	Non Verbal IQ: 78.1 (19.7)	To determine if children in these different diagnostic groups differed on rates and profiles of psychopathology	Comparative study	The ADOS^5^ The Child Behavior Checklist	Higher scores in the PDD-NOS group (school-age sample) on two items measuring Anxiety/ Depression on the Child Behavior Checklist
Davis et al.	2011	33	33	7.46 years (2.79)			USA	91.1% without ID	To examine whether or not there are fundamental differences in the effects of communication deficits on anxiety levels in children with AD and PDD-NOS	Comparative study	The Autism Spectrum Disorders – Comorbidity for Children The Autism Spectrum Disorders – Diagnostic for Children	The AD and PDD-NOS groups interacted with communication deficits such that children with AD experienced less anxiety as communication deficits increased while children with PDD-NOS experience more anxiety as communication deficits increased
Davis et al.	2010	159	154	27.09 months (5.02)			USA		To establish if there are differences in the prevalence of anxiety and avoidance between infants and toddlers diagnosed with AD versus those with PDD-NOS	Comparative study	The BISCUIT-Part 2	Results indicated an overall pattern whereby toddlers with AD had more severe anxious and avoidant symptoms than toddlers with PDD-NOS
Horovitz et al.	2010	141	135	26.68 months (4.97)			USA		To examine gender differences in psychiatric symptoms in infants and toddlers with AD and PDD-NOS	Comparative study	The BISCUIT part 2	No gender differences. The AD group evinced significantly more psychiatric symptoms (tantrum/conduct behavior, inattention/impulsivity, avoidance behavior, anxiety/repetitive behavior, eating/sleep problems) than the PDD-NOS
Matson et al.	2009	169	140	27.30 months (4.81).			USA		To examine the frequency of various comorbid problems in infants and toddlers with an AD or PDD-NOS	Comparative study	The BISCUIT-Part 2	All five disorders (tantrum/conduct behavior, inattentive/impulsive, avoidant behavior, anxiety/repetitive and eating problems/sleep) were more common in the AD group. Differences in the two groups were particularly striking for the anxiety/repetitive behavior and inattention/impulsivity factors
Gurkan et al.	2008	18	11	AD: 10.61 years (3.99) PDD-NOS: 10.27 (3.50)	11	100	Turkey	Full Scale IQ: 91.00 (23.46)	To determine the pattern and frequency of psychiatric comorbidity in children and adolescents with AD and PDD-NOS	Comparative study	The K-SADS The Wechsler Intelligence Scale for children-Revised tests	Differences were significant only for tic disorders which were highest in PDD-NOS group
Pearson et al.	2006	25	25	AD: 9.5 years (3.1) PDD-NOS: 10.5 months (3.3)	19	76	USA	Full-Scale IQ: 94.0 (22.9)	To compare differences in behavioral and emotional comorbidity in children with AD and those with PDD-NOS	Comparative study	The Personality Inventory for Children – Revised	Children with PDD-NOS were at lower risk for depression, social withdrawal, atypical behaviors/psychosis, and social skills problems and higher risk for family problems

^1^(SD). ^2^Development Quotient. ^3^Schedule for Affective Disorders and Schizophrenia for School-Age Children. ^4^Baby and Infant Screen for Children with aUtIsm Traits. ^5^Autism Diagnostic Observation Schedule.

**Table 4:** Associated physical health conditions in PDD-NOS in comparison with AD.

Authors	Year	Population		Age	Males in PDD-NOS group		Country	PDD-NOS Functioning level	Objective	Study design	Methods and main assessment tools	Results
		AD	PDD-NOS		*N*	%						
Gok et al.	2019	61	41	9.56 years (3.9)^1^			Turkey		To evaluate the relationship between sleep disturbances, gastrointestinal problems and eating behaviors in children who are diagnosed with AD and PDD-NOS	Comparative study	Parental reports on sleeping and gastrointestinal problems The Feeding Assessment Survey The Brief Autism Mealtime Behavior Inventory	Sleeping, gastrointestinal and eating problems are seen in those with AD more commonly than in those diagnosed with PDD-NOS
Carigi et al.	2014	46	71	AD: 67.78 months (39.59) PDD-NOS: 45.20 months (17.28)	56	79	Italy	DQ^2^: 59.86 (15.42)	To identify clinical features that might prove able to differentiate AD from PDD-NOS	Comparative study	Semi-structured interview to collect information about the developmental history	Sleep disorders were more frequent in the AD group.
Kozlowski et al.	2012	506	502	AD: 26.63 months (4.69) PDD-NOS: 26.35 (4.82)	376	72	USA		To compare groups of children with AD and PDD-NOS on their Eating/Sleep Problems	Comparative study	the BISCUIT^3^-Part 2	Children with AD were found to present with significantly more feeding and sleep problems than children with PDD-NOS
Mandy et al.	2011	97	66	AD: 9.43 years (3.50)^2^ PDD-NOS: 9.05 years (3.51)	52	79	UK	Verbal IQ: 87.60 (15.98)	To characterize a group of individuals diagnosed according to a clearly operationalized DSM-IV-TR definition of PDD-NOS	Comparative study	The Developmental, Dimensional and Diagnostic Interview The ADOS^4^	For sleep problems there were no group differences. By contrast, on measures of feeding difficulties the PDD-NOS group were less impaired that the AD group
Matson et al.	2009	72	40	8.21 years (3.76)			USA		To better understand the nature and specific interrelationship between feeding problems and autism and PDD-NOS should be	Comparative study	The Autism Spectrum Disorders-Diagnostic for Children The Autism Spectrum Disorders-Comorbidity for Children	Feeding problems did not differed between the AD and PDD-NOS groups
Parmeggiani et al.	2007	77	77	10 years 1 month (range: 3 years to 29 years 2 months)	48	62	Italy	ID in 92.3% of cases	To evaluate the occurrence, features, and causes of epilepsy in pervasive developmental disorder not otherwise specified in comparison with autistic disorder	Comparative study	The CARS^5^	Mild mental retardation, pathological neurological examination, cerebral lesions, abnormal EEG background activity and associated genetic pathologies were more common in PDD-NOS. Epilepsy occurred in 35.1% of patients with PDD-NOS, with no statistically significant difference compared with AD. The mean age of seizure onset was earlier (2 years 8 months) in PDD-NOS. Seizure outcome was better in AD

^1^(SD) ^2^Developmental Quotient. ^3^Baby and Infant Screen for Children with aUtIsm Traits. ^4^Autism Diagnostic Observation Schedule. ^5^Childhood Autism Rating Scale.

**Table 5:** Longitudinal studies comparing developmental trajectories in AD and PDD-NOS.

Authors	Year	Population		Age	Males in PDD-NOS group		Country	Objective	Study design	Methods	Main assessment tools	Results
		AD	PDD-NOS		*N*	%						
Usta et al.	2019	182	210	72.3 months (45.9)^1^			Turkey	To examine predictors of outcome after 3-year clinical observation, special education and psychiatric treatment in the clinical-based group	Prospective study	The ASD symptoms were assessed at baseline (T0) and 12th (T1), 24th (T2) and 36th (T3) months. The performance of machine learning algorithms was tested on the data.	The ABC^2^ The Abberant Behavior Checklist The CGI^3^	In the autism group, older father and mother age In the PDD-NOS group, mental retardation comorbidity, less birth weight and older age at diagnosis have a worse outcome
Hinnebusch et al.	2017	111	82	AD: 27.09 months (4.57) PDD-NOS: 25.88 months (4.04)	65	78	USA	To determine whether children retained an ASD diagnosis (AD or PDD-NOS) and to assess autism severity and degree of cognitive and adaptive progress	Prospective study	Children were initially evaluated at approximately 24 months of age and were then reevaluated about two years later.	The ADOS-G^4^ The CARS^5^ The VABS^6^ The Mullen Scales of Early Learning	The AD group showed a higher rate of diagnostic stability than the PDD-NOS group. The PDD-NOS group showed significanty less improvement than the AD group; however, at both time points, they were still on average in the PDD-NOS range, with lower severity scores than the AD group. No significant differences in learning rate on cognitive or adaptive measures between the AD and the PDD-NOS groups
Mordre et al.	2012	74	39	AD: 6.7 years (SD 2.6) PDD-NOS: 7.7 years (SD 2.3)	31	80	Norway	To compare adult outcome of individuals with PDD NOS and individuals with AD	Comparative study	Children with AD and PDD-NOS were followed up from childhood to adulthood. Outcome measures were criminality, mortality and marital status rates		The disability pension award was the only outcome measure that differed significantly between the AD and PDD-NOS group. The lower rate of disability pension award in the PDD-NOS group was predicted by better psychosocial functioning
Osada et al.	2012	67	31	AD: 8.99 years (0.39) PDD-NOS: 8.98 years (0.61)	24	77	Japan	To clarify how IQ, autistic symptoms, educational placements and job status vary between AD and PDD-NOS	Longitudinal comparative study	The participants’ data were collected between their first visit to the clinic and the visit at which they applied for basic disability benefits at 20 years of age.	The Japanese version of the Stanford-Binet The CARS	Participants with AD consistently showed lower IQ and more severe autistic symptoms than those diagnosed with PDD-NOS
Malhi et al.	2011	64	13	AD: 2.49 years (SD 0.41) PDD-NOS; 2.43 years (SD 0.37)			India	To describe stability and change of early diagnosis of AD and PDD-NOS	Prospective follow up study	Diagnosis of AD and PDD-NOS was made at age 3 years or less and follow-up was done around 4 years of age.	The CARS	The diagnosis of AD was more likely to persist than the diagnosis of PDD-NOS
Kim et al.	2010	121	71	8 to 30 months	63	89	USA	To study longitudinal data from toddlers and pre-schoolers (from 8 to 56 months old) with AD and PDD-NOS	Longitudinal study	A hierarchical regression analysis was carried out using children’s earlier RRB totals (from 8 to 30 months) as one of the predictors for the same children’s later RRB totals (from 31 to 56 months)	The ADOS	The likelihood of having an restrictive and repetitive behavior was the same for both AD and PDD-NOS groups; though the rated severity of these restrictive and repetitive behaviors was higher in children over age two with AD than PDD-NOS diagnoses
Anderson et al.	2009	93	51	AD: 29.6 months (4.68) PDD-NOS: 29.45 months (5.67)			USA	To examine the development of social skills between ages 2 to 13 in a sample of children initially diagnosed with AD or PDDNOS	Prospective study	Growth curve analysis was used to examine growth in socialization age equivalents from age 3 to 13 (Assessment at 2, 3, 5, 9 and 13 years)	The ADOS The ADI-R^7^ The VABS	The gap between children with AD and the PDD-NOS group widened with time as the social skills of the latter group improved at a higher rate
Chawarska et al.	2009	43	18	21.5 months (SD 4.9)			USA	To examine short-term stability of clinical diagnosis in the second year of life	Prospective study	Cognitive, social, and communication skills of toddlers were assessed at the average age of 21.5 (SD 4.9) months, and reassessed at 46.9 (SD 7.7) months	The ADOS The Mullen Scales of Early Learning	Diagnosis of AD was stable in 74% of cases as compared to 83% in PDD-NOS
van Daalen et al.	2009	40	13	AD: 29.4 (5.6) PDD-NOS: 28.2 (5.2)	9	69	The Netherlands	To evaluate the stability of ASD diagnoses in children	Prospective study	The ASD diagnoses (AD and PDD-NOS) at 23 months and 42 months of 131 patients were compared to evaluate stability of the diagnosis of ASD		The stability was 63% for AD and 54% for PDD-NOS
Anderson et al.	2007	98	58	29 months (SD 5.17)			USA	To contrast the differences between diagnostic groups in the development of verbal skills from age 2 to 9	Prospective study	Growth curve analyses were used to analyze verbal skills trajectories over time	The ADOS The ADI-R The Infant Mullen Scales of Early Learning	The gap between children with AD and the PDD-NOS group widened with time as the verbal abilities of the latter group improved at a higher rate
Takeda et al.	2007	49	77	AD: 31.7 ± 3.3 months PDD-NOS: 34.8 ± 3.5 months	39	51	Japan	To compare the change in DQ or IQ between AD and PDDNOS in preschool years	Comparative study	AD and PDD-NOS children were evaluated at age 2 and at age 5	The Kyoto Scale of Psychological Development and the Japanese version	The PDD-NOS children were significantly higher in DQ/IQ at age 2 and at age 5 than the AD children
Thurm et al.	2007	59	24	AD: 29.98 months (SD 4.28) PDD-NOS: 30.38 months (SD 4.69)	19	79	USA	To explore which early variables (e.g., non-verbal cognitive ability, Vineland social and communication skills) at ages 2 and 3, best predicted receptive and expressive language at age 5	Prospective study	The study analyses data obtained at age 2 and age 3, as well as between the ages of 4 and 5	The ADI-R The ADOS The DAS The Mullen Scales of Early Learning The VABS	VABS age equivalent ratio scores (ratio scores = age equivalent scores/chronological age) and other key variables indicated that Children with AD had significantly lower ratio scores than children with PDD-NOS on non-verbal cognitive ability and on both the socialization and communication domains of the VABS at ages 2 and 3, as well as on the composite expressive and receptive language age equivalent ratio scores at age 5
Lord et al.	2006	84	46	AD:29.1 months (SD 4.7) PDD-NOS 29.1 months (SD 5.6)	35	76	USA	To address the question of diagnostic stability of AD and PDD-NOS	Prospective study	Prospective study of diagnostic classifications made at ages 2 and 9 years	The ADI-R The ADOS	Diagnostic stability at age 9 years was very high for AD at age 2 years and less strong for PDD-NOS
Stone et al.	1999	25	12	31;4 months (SD¯3;4)			USA	To examine the stability of the diagnosis over a 1-year period	Prospective study	This sample included children who received an ASD diagnosis before 3 years and returned for a 1-year follow-up	The ADI-R The ADOS	The diagnosis of AD was more stable over time than was the diagnosis of PDD-NOS

^1^(SD). ^2^Autistic Behavior Checklist. ^3^Clinical Global Impression Severity Scale. ^4^Autism Diagnostic Observation Schedule. ^5^Child Autism Rating Scale. ^6^Vineland Adaptive Behavior Scale. ^7^Autism Diagnostic Interview – Revised.

### Cross-sectional studies

#### Autism symptoms

Studies on autism symptoms in PDD-NOS in comparison with AD deeply investigated the social-communication domain. 13 studies (33, 34, 36–46) (Table 1) showed a lesser socialization impairment whereas 3 studies (including one on gaze performance) (47–49) did not show any differences (Table 1).

7 studies found lower restrictive and repetitive behaviors (34, 35, 42, 43, 46, 50, 51) (Table 1). However, 3 studies did not show such a difference (33, 41, 52) (Table 1).

The mean age of onset of parental concerns in the group with AD was comparable to that reported in the PDD-NOS group (53). These parental concerns in PDD-NOS referred to non-specific problems linked to sleeping, eating, and level of activity (53).

#### Perinatal, developmental and functional aspects

Three studies (54–56) showed distinct perinatal risk factors in PDD-NOS in comparison with AD (Table 2). However, involved factors differed: hyperbilurinemia (55), higher exposure to smoking during pregnancy (56), threatened abortion, a labor duration of less than 1 h, cephalopelvic disproportion, and deliverance by an elective or emergency cesarean section (54). Even if perinatal risk factors might differ, odds ratios for birth defects were similar for AD and PDD-NOS (57).

Early development was less impacted in PDD-NOS with respect to AD (Table 2). Pointing was less impaired in PDD-NOS than in AD (33, 58). Skill loss was less frequent in PDD-NOS (58, 59). Children with AD began crawling significantly later than those with PDD-NOS (60). Using the Revised Educational Profile, Portoghese et al. showed that all behavioral areas and almost all assessed developmental domains (with the exception of the cognitive verbal) were more severely impaired in the AD group than in the PDD-NOS group (61). Children with AD and ID displayed a Visual Rooting Reflex more often than children with PDD-NOS and ID (62), suggesting less severe neurological impairments.

The investigation of language development in PDD-NOS in comparison with AD did not show any difference or particularity (63, 64) (Table 2). Also, fine and gross motor development did not differ between the 2 groups (35, 65). However, Ghaziuddin et al. found lower clumsiness in PDD-NOS (66).

Regarding socio-emotional development, social cognitive performance did not differ between both groups (67). One study shows a distinct profile of emotional information treatment in PDD-NOS in comparison with AD (68) (Table 2).

Children with PDD-NOS and AD did not differ with respect to global intellectual functioning (35, 69, 70). However, one study (35) showed higher visio-spatial performance in PDD-NOS when others did not (71, 72) (Table 2).

Executive functions lead to contradictory results, showing lesser impairment in PDD-NOS (73, 74) or no difference (75) (Table 2).

Sensory profile did not differ between the two groups (76).

Adaptive function was higher in PDD-NOS in 2 studies (34, 77). However, other studies did not show any difference (78, 79) (Table 2).

Quality of life of children and parents was less impaired in the PDD-NOS groups (33, 80) (Table 2).

Only one study showed an impact of sex on AD vs. PDD-NOS (81).

#### Associated mental health conditions

Children with PDD-NOS showed less challenging behavior than those with AD (82–86). Symptoms related to ADHD were overrepresented in the AD group (38, 85) (Table 3). Studies showed more Conduct Disorder (87) and Oppositional Defiant Disorder (88) in the PDD-NOS group.

Anxiety led to contradictory results (Table 3). Anxiety has been shown to be higher in the AD group (83, 85, 89) or in the PDD-NOS group (90). A study investigated the interaction between diagnosis (AD or PDD-NOS) and communication deficits (91). Children with AD experienced less anxiety as communication deficits increased while children with PDD-NOS experience more anxiety as communication deficits increased highlighting distinct type of interaction (91).

Chien et al. showed that children with PDD-NOS had a higher risk for developing Schizophrenia Spectrum Disorder, Bipolar Disorder, Major Depressive Disorder than those with AD (92). These results concur with another study comparing youth with ASD attending a specialty clinic to those attending a general psychiatry clinic (88). The latter group suffered from a greater burden of psychopathologies (Major Depressive Disorder and psychosis), higher levels of dysfunction and more pharmacotherapy [*cf* Carigi et al. (33); Table 3].

#### Associated physical health conditions

Studies showed less sleep disorders in the PDD-NOS-group (33, 93, 94) but another did not (35) (Table 4). Feeding disorders were less common in the PDD-NOS group than in the AD group (35, 93, 94). However, one study did not confirm this result (85) (Table 4).

Parmeggiani et al. showed that pathological neurological examination, cerebral lesions, abnormal EEG background activity and associated genetic pathologies were more common in PDD-NOS. Moreover, Epilepsy seizure in PDD-NOS had an earlier onset (2 years 8 months) and a better outcome (95).

### Longitudinal studies

Longitudinal studies showed lower diagnosis stability in PDD-NOS than in AD (27, 96–99) with the exception of Chawaska et al. study (100) (Table 5).

Cognitive development was less impaired in PDD-NOS (Osada et al., Takeda et al., Thurm et al.) (101–103). However, another study did not show such a better outcome in this group (96).

As a whole, autistic symptoms were lower in the PDD-NOS group than in the AD group (96, 101) (Table 5). More specifically, studies showed better communication and socialization skill outcomes in PDD-NOS than in AD (103–105). Kim et al.’s study showed that the likelihood of having restrictive and repetitive behavior was the same for both AD and PDD-NOS groups; though the rated severity of these restrictive and repetitive behaviors was higher in children over age 2 with AD than with children with a PDD-NOS diagnosis (106).

Regarding functional outcome, a study form Norway (107) showed that the disability pension award at adulthood was the only outcome measure differing significantly between the AD and PDD-NOS groups previously included during childhood.

Within the PDD-NOS group, worse outcome was associated with ID, lesser birth weight and older age at diagnosis (108). Moreover, a better psychosocial functioning during childhood predicted a lower rate of disability pension award in the PDD-NOS group (107).

## Discussion

We conducted a systematic review of PDD-NOS in comparison with AD. Cross-sectional and longitudinal studies comparing PDD-NOS and AD showed a clear trend for lower communication and socialization impairment in children with PDD-NOS. Results on restrictive and repetitive behaviors were more contradictory. Early development was less impacted in PDD-NOS as well as the quality of life of children and families. Children with PDD-NOS showed less challenging behavior than those with AD. Schizophrenia and mood disorders appeared to be higher in PDD-NOS whereas feeding and sleep disorders tend to be less prevalent.

With regard to our results, PDD-NOS appeared as category both quantitatively and qualitatively distinct from AD. In a quantitative perspective, PDD-NOS was a lesser variant of autism with respect to the trajectory of autism symptoms, some associated mental and physical health conditions, or quality of life. This perspective is consistent with the spectrum approach of the DSM-5 (2) and a possible continuum between AD and PDD-NOS within ASD. However, in a qualitative perspective, PDD-NOS differed from AD because of higher associated schizophrenia and mood disorders. These results were unexpected with respect to a spectrum approach and suggest specific links between the margins of autism and other psychiatric dimensions. Our review coheres with Tsai and Ghaziuddin’s review (109) which concluded that the literature appeared to suggest that PDDNOS can be separated from AD, and, therefore, does not seem to lie on a “continuum” with that disorder. These authors recommended systematic research to settle the issue and that PDDNOS should be regarded as a separate category to facilitate ongoing research (109). In contrast, a recent systematic review on PDD-NOS (110) concluded that in regard of the limited consistency, sensibility, and stability of this disorder, it would be more consistent to include this diagnosis within ASD. Noticeably, the authors still recommended to collect information from professionals on adults who received a PDD-NOS diagnosis in childhood.

Our search on PubMed found 510 references. On the same period, from 1987 to 2022, the term “Autistic disorder” gave 24,639 references. PDD-NOS was thus referenced 48 times less than AD, and was therefore much less studied. However, the high prevalence of the PDD-NOS category demonstrates its high clinical relevance. This clinical importance of PDD-NOS in psychiatry was also emphasized by a study comparing youth with PDDs attending a specialty clinic for autism with those attending a general psychiatry clinic (88). More autism clinic youth met criteria for AD; more of those referred to the psychiatry clinic met the criteria for PDD-NOS (88). The discrepancy between the past clinical use of the PDD-NOS category and the scarcity of research in the field (in comparison with AD) might be due to the difficulty of studying the margins of autism because of their heterogeneity and complexity - which our review aimed to underline. PDD-NOS therefore illustrated a gap between clinical and research issues and the need to address heterogeneity from a research perspective. Based on research rather than clinical consideration of the PDD-NOS, the DSM-5 missed the complexity of the margins and opted for a continuum approach and ASD.

With regard to the 4 recommendations proposed by Towbin on the use of the PDD-NOS diagnosis category (21), it appears that, firstly, although PDD-NOS is related to earlier developmental disorders, the age of parental concern does not differ between AD and PDD-NOS. The latter result suggests that the PDD-NOS is not commonly used in the case of a later age of onset of autistic symptoms. Towbin (21) and Mandy et al. (35) suggested that PDD-NOS depicted a clinical picture with early onset of symptoms, an impairment in social reciprocity and less to no restrictive and repetitive behaviors. We showed, however, the lack of clear support to the claim of lesser frequency of restrictive and repetitive behaviors in PDD-NOS. Towbin also recommended using PDD-NOS as a temporary diagnosis when data are lacking or when the child is too young (21). This recommendation concurs with less diagnostic stability in PDD-NOS in infants and pre-schoolers, as is shown by our results. Finally, our review mostly supports the recommendation referring to PDDS-NOS as a lesser variant of autism with respect to the trajectory of autism symptoms, associated mental and physical health conditions, or quality of life.

Chien et al. showed that PDD-NOS was more associated than AD with schizophrenia and mood disorder (92). This result concurs with Joshi et al.’s study comparing youth with PDDs attending a specialty clinic for autism to those attending a general psychiatry clinic (88). General psychiatry clinic youth with PDDs (with more PDD-NOS than AD) suffered from more Major Depressive Disorder and psychosis. This result echoes the results of the Sporn et al.’s retrospective study, showing that 25% of the 76 patients who reported early onset schizophrenia had a diagnosis of PDDs during their childhood, with a clear predominance of PDD-NOS (111). In a reference sample of childhood onset schizophrenia, Rapaport et al. also found a high frequency of PDD-NOS (112).

The importance of associated psychosis and mood disorder in PDDs also echoes past syndromic conceptualization such as Multiple Complex Developmental Disorder (MCDD) (113), Pathological Demand Avoidance (PDA) (114) or Multi-Dimensional Impairments (MDI) (115) which linked social impairment, emotional lability and signs of psychosis (without a diagnosis of early onset schizophrenia). These signs of psychosis referred to thought disorder (MCDD), delusions (MCDD), poor ability to distinguish fantasy from reality (MDI, PDA). It is important to stress that MCDD and PDA were initially coined to deal with heterogeneity within PDD-NOS. However, both syndromes were not limited to the PDD-NOS category (116, 117).

The results of our review highlight that PDD-NOS was of great interest because of its high prevalence and clinical relevance. This diagnosis was useful to take into account milder forms of autism. However, it was less reliable and less stable through time than AD. Moreover, a qualitative study emphasized (118) that the autistic community might not acknowledge this diagnosis. This study was a phenomenological analysis of discussion forum dialog among 76 adult participants with self-reported diagnosis of AD and AS but not PDD-NOS (118). Participants constantly agreed that PDD-NOS should be removed in the DSM-5. Several participants expressed concern that the PDD-NOS diagnosis was not specific and reliable enough. They called the diagnosis “a junk category,” “a receptacle” (118).

Our review emphasized lower socio-communication impairments in PDD-NOS than in AD. In DSM-5, ASD diagnosis required three criteria out of three in this domain, namely deficits in social–emotional reciprocity, deficits in nonverbal communicative behaviors used for social interaction and deficits in developing, maintaining, and understanding relationship (2). To diagnose PDD-NOS, only two of them was required (18). It might therefore be suspected that the social and communication deficit characteristic of PDD-NOS wasn’t important enough for it to remain in the autism spectrum. A review and meta-analysis (119) showed that 70% of PDD-NOS did not meet the criteria for ASD. Another hypothesis postulated that PDD-NOS might belong to the Social Communication Disorder (SCD) diagnosis category. However, this category must meet all three criteria for the social-communication domain, as in ASD (2). Thus, it appears that PDD-NOS has been excluded from ASD and SCD.

Our review challenged initial views of PDD-NOS and showed that this diagnosis mostly included milder forms of autism. PDD-NOS was very clinically relevant to deal with the margins of autism and the diversity within the spectrum. However, PDD-NOS encountered too many limitations (low reliability, instability through time, low acceptability) to be retained in the DSM-5. The fact is that many PDD-NOS did not join either ASD or SCD. Thus, in order to take into account past research on PDD-NOS and the margin of autism, we argue for the creation of a new category alongside ASD and SCD, based on three main dimensions related to socialization impairment, emotional lability and psychotic symptoms and coined on PDA, MCDD and MDI. Criteria for this new category could be selected from the most valid criteria for these three syndromes and tested against the ASD criteria, to ensure that they are truly distinct. In our view, this new category would provide an opportunity for mental health professionals, along with SCD and ASD, to better take into account the neurodevelopmental disorders that have an impact on the development of social communication and its heterogeneity. From a clinical point of view, this new category would trigger the search for associated psychotic symptoms or emotional lability that might currently be understudied by professionals. In addition, new developmental trajectories, specific interventions and treatments could be identified, which would in turn make it possible to better inform and help the people concerned and their families.

## Limitations

The first limitation of our study comes from the terms selected for the database search. Few studies directly addressed differences between PDD-NOS and AD. However, when the DSM-IV-TR was in use, some studies included participants with PDDs including those with AD, PDD-NOS or another PDD subtype often compared to controls. In the latter case, PDD-NOS was only mentioned in the abstract or in the title when there was a difference within PDDs subgroups. That is why we may have over-selected references showing a difference between PDD-NOS and AD, and under-selected negative studies that did not. The second limitation is that we studied all clinical features of PDD-NOS. For example, some characteristics such as quality of life were rarely studied and were only related to two references. The third limitation is sample overlap between studies, as 25% of the studies were from Matson et al. Finally, our results cannot be extended to females with PDD-NOS, individuals with PDD-NOS and ID, or adults with PDD-NOS.

## Conclusion

Our systematic review shows that PDD-NOS corresponds to milder form of autism with less impact and less associated disorder, with the exception of schizophrenia and mood disorder. Our review challenges initial views of PDD-NOS, echoes past syndromic conceptualizations such as MCDD, PDA or MDI, and argues for a quantitative and qualitative distinction between AD and PDD-NOS. The PDD-NOS diagnosis was very clinically relevant to deal with the margins of autism and the diversity within the spectrum, and therefore massively used by professionals in the field. However, PDD-NOS had many limitations (low reliability, instability through time, low acceptability) and was therefore understudied, thereby generating a discrepancy between clinical and research issues. Thus, in order to overcome this gap and to take into account past research on PDD-NOS, we suggest taxonomic changes in DSM-5, through the introduction of a new category based on three main dimensions: socialization impairment, emotional lability and psychotic symptoms. Future studies are therefore needed to test relevant criteria for this new category and possibly identify developmental trajectories, specific interventions and treatments.

## Author contributions

RC and OP designed the systematic review and selected the studies following PRISMA guidelines. OP was contributed mainly in epistemological and methodological. RC wrote the first complete draft of the manuscript. OP, NC, and CS provided substantial modification to the manuscript. All authors contributed to the article and approved the submitted version.
